# Training tomorrow's doctors in diabetes: self-reported confidence levels, practice and perceived training needs of post-graduate trainee doctors in the UK. A multi-centre survey

**DOI:** 10.1186/1472-6920-8-22

**Published:** 2008-04-17

**Authors:** Jyothis T George, David A Warriner, Jeffrin Anthony, Kavitha S Rozario, Sinu Xavier, Edward B Jude, Gerard A McKay

**Affiliations:** 1 York Hospital, York, UK; 2 Good Hope Hospital, Birmingham, UK; 3 Harrogate Health Care, Harrogate, UK; 4 University Hospital, Walsgrave, UK; 5 Tameside General Hospital, Ashton-under-Lyne, UK; 6 Glasgow Royal Infirmary, Glasgow, UK

## Abstract

**Objective:**

To assess the confidence, practices and perceived training needs in diabetes care of post-graduate trainee doctors in the UK.

**Methods:**

An anonymised postal questionnaire using a validated 'Confidence Rating' (CR) scale was applied to aspects of diabetes care and administered to junior doctors from three UK hospitals. The frequency of aspects of day-to-day practice was assessed using a five-point scale with narrative description in combination with numeric values. Respondents had a choice of 'always' (100%), 'almost always' (80–99%), 'often' (50–79%), 'not very often' (20–49%) and 'rarely' (less than 20%). Yes/No questions were used to assess perception of further training requirements. Additional 'free-text' comments were also sought.

**Results:**

82 doctors completed the survey. The mean number of years since medical qualification was 3 years and 4 months, (range: 4 months to 14 years and 1 month). Only 11 of the respondents had undergone specific diabetes training since qualification.

4(5%) reported 'not confident' (CR1), 30 (37%) 'satisfactory but lacked confidence' (CR2), 25 (30%) felt 'confident in some cases' (CR3) and 23 (28%) doctors felt fully confident (CR4) in diagnosing diabetes. 12 (15%) doctors would always, 24 (29%) almost always, 20 (24%) often, 22 (27%) not very often and 4 (5%) rarely take the initiative to optimise gcaemic control. 5 (6%) reported training in diagnosis of diabetes was adequate while 59 (72%) would welcome more training. Reported confidence was better in managing diabetes emergencies, with 4 (5%) not confident in managing hypoglycaemia, 10 (12%) lacking confidence, 22 (27%) confident in some cases and 45 (55%) fully confident in almost all cases. Managing diabetic ketoacidosis, 5 (6%) doctors did not feel confident, 16 (20%) lacked confidence, 20 (24%) confident in some cases, and 40 (50%) felt fully confident in almost all cases.

**Conclusion:**

There is a lack of confidence in managing aspects of diabetes care, including the management of diabetes emergencies, amongst postgraduate trainee doctors with a perceived need for more training. This may have considerable significance and further research is required to identify the causes of deficiencies identified in this study.

## Background

Prevalence of diabetes in adults worldwide was estimated to be 4.0% in 1995 and to rise to 5.4% by the year 2025. [1] In the UK, diabetes is presently diagnosed in over two million people [2] and is set to rise [3]. People with diabetes develop a wide range of medical and surgical problems [4], [5]. Therefore, irrespective of their eventual specialty, trainee doctors are likely to be involved in the care of people with diabetes once they complete their post-graduate training.

Multiple therapeutic interventions for preventive care make these patients increasingly complex to manage. It is common for patients to be on multiple tablets to address blood glucose control [6] and Insulin is often combined with oral hypoglycaemic agents [7]. There is an ever-increasing evidence-base recommending good control of blood pressure, lipids, body mass index and interventions such as smoking cessation, alcohol reduction, increased levels of exercise and the use of anti-platelet treatment to ensure optimum preventive care for people with diabetes [[4,8-10] and [11]]. As a result, doctors caring for people with diabetes, particularly those responsible for their long-term care have to deal with poly-pharmacy, the potential for adverse drug interactions and the need to constantly re-balance the risks and benefits of therapeutic options as more evidence emerges [12]. Therefore, it is important that all trainee doctors acquire adequate knowledge and skills in the management of diabetes.

There are many potential barriers to the acquisition of knowledge and skills in management of diabetes by trainee doctors [13], with the proposed changes in the duration and structure of post-graduate training raising [14] further challenges. We were unable to find any studies in the literature to clarify the extent of this problem and sought in this pilot survey to establish the self-confidence, practices and perceived training needs of trainee doctors in aspects of diabetes care.

## Methods

A pre-validated anonymised questionnaire was delivered by post (single mailing) to trainee doctors in three different centres in the UK (2 in England, 1 in Scotland) with covering letters from the local investigators inviting them to participate.

### Sample

The target sample was defined as all post graduate trainees in general (internal) medicine and related specialties including emergency medicine and intensive care medicine. Specialist trainees in diabetes and endocrinology were excluded. This cohort with day-to-day exposure to the theory and practice of diabetes care provides a representative sample to assess current practice and training. We consulted the local ethics committee was consulted and formal application was considered not necessary. All respondents consented to the use of their responses for analysis. We aimed to approach two hundred trainees expecting a 30% response rate.

### The questionnaire

To assess self-reported confidence among junior doctors, we used the 'Confidence Rating' (CR) scale used by the Royal College of Physicians in the self-assessment of trainees [15]. The scale has four points – ('not confident' (CR1), 'satisfactory but lacking confidence' (CR2), 'confident in some cases (CR3) and 'fully confident in most cases' (CR4). The option to provide additional 'free-text' comments was also provided. To assess the frequency of aspects of day-to-day practice, we used a five-point scale with narrative description in combination with numeric values. Respondents had a choice of 'always' (100%), 'almost always' (80–99%), 'often' (50–79%), 'not very often' (20–49%) and 'rarely' (less than 20%).

The questionnaire was validated in a four-stage process: Initial review by external experts in the field of diabetes, administration of initial draft on a sample cohort, revision of questionnaire based on feedback and final external review.

### Analysis of results

Self-reported answers were tabulated and expressed as number (n) and in percentages rounded off to the next percentage point.

## Results

### Demographics

82 doctors returned the completed survey, achieving a 41% response rate with 200 questionnaires sent out. 19 were house officers or Foundation Year 1 doctors, 48 were senior house officers or Foundation Year 2 doctors, 11 were specialist registrars and four did not specify their stage of training. The mean number of years since medical qualification was 3 years and 4 months, (range: 4 months to 14 years and 1 month).

Only 10 of the respondents had undergone specific diabetes training since qualification. 35 of the doctors had never worked in teams lead by specialists in Diabetes, the rest on average having worked for 10 weeks, with a maximum of 3 years.

### Confidence levels

In diagnosing diabetes, 4 (5%) reported they were 'not confident' (CR1), 30 (36%) 'satisfactory but lacked confidence' (CR2), 25 (31%) felt 'confident in some cases' (CR3) and 23 (28%) doctors felt fully confident (CR4). Table 1 details self-reported confidence levels in diagnosis and management of emergencies. Confidence levels in diagnosing retinopathy and the use of intravenous Insulin are tabulated in table 2. There is some evidence of increasing confidence levels in more senior training grades.

**Table 1:** Self-reported confidence levels in diagnosis and management of emergencies (n = 82).

	**CR1**	**CR2**	**CR3**	**CR4**	**No Answer**	**Total**
**Diagnosing Diabetes**						
**All respondents (n = 82)**	**4 (5%)**	**30 (37%)**	**25 (30%)**	**23 (28%)**	**0**	**82 (100%)**
House Officers (n = 19)	0	10 (53%)	7 (37%)	2 (11%)	0	19 (100%)
Senior House Officers(n = 48)	3 (6%)	17 (35%)	14 (29%)	14 (29%)	0	48 (100%)
Specialist Registrars (n = 11)	0	2 (18%)	3 (27%)	6 (55%)	0	11 (100%)
Others(n = 4)	1(25%)	0	1(25%)	2(50%)	0	4(100%)
**Diagnosing IGT**						
**All respondents (82)**	**16 (20%)**	**24 (29%)**	**24 (29%)**	**18 (22%)**	**0**	**82 (100%)**
House Officers (n = 19)	6 (32%)	6 (32%)	5 (26%)	2 (11%)	0	19 (100%)
Senior House Officers(n = 48)	8 (17%)	16 (33%)	14 (29%)	10 (21%)	0	48 (100%)
Specialist Registrars (n = 11)	0	1 (9%)	4 (36%)	6 (55%)	0	11 (100%)
Others(n = 4)	2(50%)	1(25%)	1(25%)	0	0	4(100%)
**Hypoglycaemia**						
**All respondents (82)**	**4 (5%)**	**10 (12%)**	**22 (27%)**	**45 (55%)**	**1 (1%)**	**82 (100%)**
House Officers (n = 19)	3 (16%)	5 (26%)	9 (47%)	2 (11%)	0	19 (100%)
Senior House Officers(n = 48)	0	4 (8%)	13 (27%)	30 (63%)	1 (2%)	48 (100%)
Specialist Registrars (n = 11)	0	1 (9%)	0	10 (91%	0	11 (100%)
Others(n = 4)	1(25%)	0	0	3 (75%)	0	4(100%)
**Diabetic Ketoacidosis**						
**All respondents (82)**	**5 (6%)**	**16 (20%)**	**20 (24%)**	**40 (49%)**	**1 (1%)**	**82 (100%)**
House Officers (n = 19)	4 (21%)	8 (42%)	6 (32%)	1 (5%)	0	19 (100%)
Senior House Officers(n = 48)	1 (2%)	5 (10%)	12 (25%)	29 (60%)	1 (2%)	48 (100%)
Specialist Registrars (n = 11)	0	2 (18%)	2 (18%)	7 (64%)	0	11 (100%)
Others(n = 4)	0	1(25%)	0	3 (75%)	0	4 (100%)
**Hyperosmolar state**						
**All respondents (82)**	**18 (22%)**	**14 (17%)**	**32(39%)**	**17 (21%)**	**1 (1%)**	**82 (100%)**
House Officers (n = 19)	10 (53%)	4 (21%)	3 (16%)	1 (5%)	1 (5%)	19 (100%)
Senior House Officers(n = 48)	5 (10%)	10 (21%)	22 (46%)	11 (23%)	0	48 (100%)
Specialist Registrars (n = 11)	2 (18%)	0	5 (45%)	4 (36%)	0	11 (100%)
Others(n = 4)	1(25%)	0	2(50%)	1(25%)	0	4 (100%)

CR1 = not confident; CR2 = 'satisfactory but lacking confidence; CR3 = confident in some cases and CR4 = fully confident in most cases. n = 82. Results expressed as n (%)

**Table 2:** Self-reported confidence levels in diagnosing retinopathy and the use of intravenous insulin.

	**CR1**	**CR2**	**CR3**	**CR4**	**No Answer**	**Total**
**Diagnosing Retinopathy**						
**All respondents (82)**	**42 (51%)**	**22 (27%)**	**11 (13%)**	**5 (6%)**	**2 (2%)**	**82 (100%)**
House Officers (n = 19)	9 (47%)	4 (21%)	3 (16%)	1 (5%)	2 (11%)	19 (100%)
Senior House Officers(n = 48)	28 (58%)	14 (29%)	4 (8%)	2 (4%)	0	48 (100%)
Specialist Registrars (n = 11)	4 (36%)	1 (9%)	4 (36%)	2 (18%)	0	11 (100%)
Others(n = 4)	1(25%)	3 (75%)	0	0	0	4 (100%)
**Commencing IV insulin**						
**All respondents (82)**	**15 (18%)**	**20 (24%)**	**18 (22%)**	**29 (35%)**	**0**	**82 (100%)**
House Officers (n = 19)	7 (37%)	7 (37%)	3 (16%)	2 (11%)	0	19 (100%)
Senior House Officers(n = 48)	6 (13%)	11 (23%)	12 (25%)	19 (40%)	0	48 (100%)
Specialist Registrars (n = 11)	1 (9%)	1 (9%)	3 (27%)	6 (55%)	0	11 (100%)
Others(n = 4)	1(25%)	1(25%)	0	2(50%)	0	4 (100%)
**Titrating IV insulin**						
**All respondents (82)**	**14 (17%)**	**24 (29%)**	**26 (32%)**	**18 (22%)**	**0**	**82 (100%)**
House Officers (n = 19)	8 (42%)	7 (37%)	3 (16%)	1 (5%)	0	19 (100%)
Senior House Officers(n = 48)	4 (8%)	14 (29%)	19 (40%)	11 (23%)	0	48 (100%)
Specialist Registrars (n = 11)	1 (9%)	1 (9%)	4 (36%)	5 (45%)	0	11 (100%)
Others(n = 4)	0	1(25%)	1(25%)	2(50%)	0	4 (100%)
**Prescribing IV fluids**						
**All respondents (n = 82)**	**4 (5%)**	**21 (26%)**	**26 (32%)**	**30 (36%)**	**1 (1%)**	**82 (100%)**
House Officers (n = 19)	0	10 (53%)	7 (37%)	2 (11%)	0	19 (100%)
Senior House Officers(n = 48)	4 (8%)	9 (19%)	17 (35%)	17 (35%)	1 (2%)	48 (100%)
Specialist Registrars (n = 11)	0	1 (9%)	1 (9%)	9 (82%)	0	11 (100%)
Others(n = 4)	0	1(25%)	1(25%)	2(50%)	0	4 (100%)

n = 82. CR1 = not confident; CR2 = 'satisfactory but lacking confidence; CR3 = confident in some cases and CR4 = fully confident in most cases. n = 82. Results expressed as n (%)

n = 8, results shown as n (%)

There is a noticeable difference in confidence levels among those respondents (n = 10) who have had specific post graduate training in diabetes, with 50 to 90 percent reporting "fully confident" (CR4) across domains. 18 to 51% of trainees without such training (n = 72) achieved "fully confident" (CR4) levels.

### Day-to-day practice

When asked if they would take the initiative to control blood glucose for their patients, 12 (15%) doctors would always take the initiative, 24 (29%) almost always, 20 (24%) often, 22 (27%) not very often and 4 (5%) rarely.

Self-reported frequencies of interventions to improve glycaemic control in day-to-day practice in are given in table 3. Frequency of identifying diabetic complications and cardiovascular risk factors are summarised in table 4.

**Table 3:** Frequency of interventions to improve glycaemic control.

	Always	Almost Always	Often	Not very often	Rarely	No response
**Educating patients on lifestyle**						
**All respondents (82)**	9 (11%)	27 (33%)	22 (27%)	18 (22%)	6 (7%)	0
House Officers (n = 19)	1 (5%)	4 (21%)	6 (32%)	6 (32%)	2 (11%)	0
Senior House Officers(n = 48)	5 (10%)	17 (35%)	14 (29%)	8 (17%)	4 (8%)	0
Specialist Registrars (n = 11)	1 (9%)	6 (55%)	1 (9%)	3 (27%)	0	
Others(n = 4)	2(50%)	0	1(25%)	1(25%)	0	0
**Changing insulin doses/timing**						
**All respondents (82)**	6 (7%)	12 (15%)	20 (24%)	31(38%)	13 (16%)	0
House Officers (n = 19)	0	3 (16%)	1 (5%)	10 (53%)	5 (26%)	0
Senior House Officers(n = 48)	5 (10%)	5 (10%)	15 (31%)	16 (33%)	7 (15%)	0
Specialist Registrars (n = 11)	1 (9%)	3 (27%)	2 (18%)	4 (36%)	1 (9%)	0
Others(n = 4)	0	1(25%)	2(50%)	1(25%)	0	0
**Changing insulin type and or device**						
**All respondents (82)**	1(1%)	4(5%)	4(5%)	30(37%)	43(52%)	0
House Officers (n = 19)	0	1 (5%)	0	7 (37%)	11 (58%)	0
Senior House Officers(n = 48)	0	2 (4%)	3 (6%)	17 (35%)	26 (54%)	0
Specialist Registrars (n = 11)	1 (9%)	1 (9%)	1 (9%)	4 (36%)	4 (36%)	0
Others(n = 4)	0	0	0	2(50%)	22(50%)	0
**Commencing/changing tablets**						
**All respondents (82)**	1(1%)	17(20%)	20 (24%)	31(39%)	12 (15%)	1(1%)
House Officers (n = 19)	0	3 (16%)	4 (21%)	6 (32%)	6 (32%)	0
Senior House Officers(n = 48)	0	11 (23%)	12 (25%)	20 (42%)	4 (8%)	1 (2%)
Specialist Registrars (n = 11)	1 (9%)	2 (18%)	3 (27%)	4 (36%)	1 (9%)	0
Others(n = 4)	0	1(25%)	1(25%)	1(25%)	1(25%)	0

(n = 82). Results shown as n (%).

**Table 4:** Frequency of identification diabetic complications in daily practice

	**Always**	**Almost Always**	**Often**	**Not very often**	**Rarely**	**No response**
**Identifying cardiovascular risk factors**						
**All respondents (82)**	**15 (18%)**	**37 (45%)**	**15 (19%)**	**9 (11%)**	**5 (6%)**	**1(1%)**
House Officers (n = 19)	3 (16%)	10 (53%)	4 (21%)	1 (5%)	1 (5%)	0
Senior House Officers(n = 48)	8 (17%)	20 (42%)	10 (21%)	7 (15%)	2 (4%)	1 (2%)
Specialist Registrars (n = 11)	2 (18%)	5 (45%)	1 (9%)	1 (9%)	2 (18%)	0
Others(n = 4)	0	2(50%)	0	0	2(50%)	
**Identifying feet complications**						
**All respondents (82)**	**15 (18%)**	**17(21%)**	**24(29%)**	**17(21%)**	**9(11%)**	**0**
House Officers (n = 19)	5 (26%)	5 (26%)	7 (37%)	1 (5%)	1 (5%)	0
Senior House Officers(n = 48)	5 (10%)	9 (19%)	12 (25%)	15 (31%)	7 (15%)	0
Specialist Registrars (n = 11)	2 (18%)	3 (27%)	4 (36%)	1 (9%)	1 (9%)	0
Others(n = 4)	2(50%)	0	0	0	0	2(50%)
**Identifying diabetic nephropathy**						
**All respondents (82)**	**17 (21%)**	**21 (25%)**	**23 (28%)**	**16 (20%)**	**5 (6%)**	**0**
House Officers (n = 19)	4 (21%)	4 (21%)	5 (26%)	5 (26%)	1 (5%)	0
Senior House Officers(n = 48)	9 (19%)	11 (23%)	15 (31%)	9 (19%)	2 (4%)	2 (4%)
Specialist Registrars (n = 11)	1 (9%)	4 (36%)	3 (27%)	2 (18%)	1 (9%)	0
Others(n = 4)	2(50%)	0	0	0	0	2(50%)
**Identifying eye complications**						
**All respondents (82)**	**6 (7%)**	**15 (18%)**	**15 (18%)**	**28 (35%)**	**18 (22%)**	**0**
House Officers (n = 19)	2 (11%)	2 (11%)	2 (11%)	6 (32%)	7 (37%)	0
Senior House Officers(n = 48)	2 (4%)	10 (21%)	8 (17%)	19 (40%)	9 (19%)	0
Specialist Registrars (n = 11)	1 (9%)	2 (18%)	4 (36%)	2 (18%)	2 (18%)	0
Others(n = 4)	1(25%)	0	0	1(25%)	0	2(50%)

### Perceived training needs

Only five of our respondents felt their training in diagnosis of diabetes and impaired glucose tolerance was adequate. 59 (72%) would welcome more training in this area. 46 (56%) of our respondents perceive further training to be required in managing diabetes emergencies as well as educating patients.

55 (67%) required further training in diagnosis of complications of diabetes while 60 (74%) required further training in modifying diabetes treatment. 46 (56%) reported a requirement for further training in the use of intravenous insulin. 15–18 (18–22%) did not give an answer to these questions on perceived training needs. Other self-reported learning needs are summarised in table 5.

**Table 5:** Self-reported training needs (n = 82)

**Is further training required in this particular area?**	**Yes**	**No**	**No Answer**
**Diagnosing diabetes and impaired glucose tolerance**	**59 (72%)**	**7 (9%)**	**16 (19%)**
Managing Diabetic Emergencies	46 (56%)	18 (22%)	18 (22%)
**Educating Patients with diabetes**	46 (56%)	20 (24%)	16 (20%)
Modifying treatment for diabetes	60 (73%)	6 (7%)	16 (20%)
**Diagnosing complications of diabetes**	55 (67%)	12 (15%)	15 (18%)
Using intravenous insulin	46 (56%)	19 (23%)	17 (21%)
**Involving diabetes specialists (nurses/doctors)**	41 (50%)	26 (32%)	15 (18%)

There was no noticeable difference in self-reported training needs of doctors from different training grades. A clear message from the free text comments by respondents was the need for more training.

## Discussion

Although the NHS does not lay down standards to benchmark expected knowledge and competencies in diabetes, there appears to be a shortfall.

With 40% of our respondents lacking confidence to diagnose diabetes, identifying the estimated half a million undiagnosed people in the UK [2] would continue to be a challenge. A quarter of doctors responding to this survey would not take the main initiative to control blood glucose for a patient under their care in hospital. This would be of concern, as hyperglycaemia is known to adversely affect outcomes in various illnesses [16,17]. It is now well established that control of blood pressure, lipid-lowering therapy and other risk factors can reduce the cardio-vascular risk in people with diabetes [18]. It is therefore disappointing to note that a fifth of our respondents would not actively identify these risk factors in people with diabetes, with lack of training cited as a common reason.

With the high prevalence of people with diabetes in hospitals [16,19], it is important for health professionals across different specialties to be confident in the management of acutely unwell patients with diabetes. However, a quarter of our respondents reported lack of confidence in management of diabetic ketoacidosis, a potentially fatal complication of diabetes. For emergencies with high mortality like ketoacidosis, it is important to have high levels of skills, knowledge and confidence amongst frontline junior medical staff.

Confidence was also reported to be low in commencing intravenous insulin, titrating intravenous insulin, prescribing intravenous fluids and changing diabetes regime. Our results show doctors are relying on specialist diabetes teams, especially specialist nurses for management of people with diabetes. Though this may help on the short term, it is likely to lead to further erosion of skills, knowledge and confidence among junior doctors. Moreover, many hospitals are yet to establish inpatient specialist nurse service [20] and concerns about deficiencies in delivery of care for people with diabetes remain. [21]

In the UK, there is a trend towards moving long-term management of diabetes from specialist centres to primary care [22]. This has also coincided with a decrease in length of training for doctors in both primary and secondary care. [14] It is therefore not surprising that close to three quarters of our sample express an appetite for further training in relatively fundamental aspects of diabetes, irrespective of experience. A comparative study of knowledge (general practitioners versus medical students), concluded a post-graduate course would be useful, at least for general practitioners [23]. With medical students completing training also reporting concerns about deficiencies in training [24], our survey highlights the necessity for focussed postgraduate training in diabetes. Although postgraduate training opportunities in diabetes exist, widespread uptake is currently unlikely because it is not built into mainstream post-graduate curriculum.

Appointment to traditional House Officer, Senior House Officer and Specialist Registrar training programs ceased in 2007 [14]. When the recommendations of MMC Inquiry Panel [25] are implemented, graduate doctors will have a one year foundation programme followed by core specialty of around three years and higher specialist training of at least three years. The curriculum and structure for these programs are being revised, presenting educators with an opportunity to include knowledge and skills in diabetes care as a core component of post-graduate training. In this pilot study, we report results based on a limited number of trainees responding to our questionnaire. However, we believe the findings are important and need to be addressed, because of concerns about confidence and practice in diabetes care amongst trainee doctors in the UK. Further research is required to quantify the extent of the problem areas identified in this pilot study.

## Conclusion

Our study demonstrates a lack of confidence in managing many aspects of diabetes care amongst post-graduate trainee doctors, including the management of diabetes emergencies where mortality may be high. Many trainees perceive a need for more training in all aspects of diabetes care, suggesting a need for structured post-graduate training in diabetes. Changes proposed to medical training by the Modernising Medical Careers Enquiry Panel present a unique opportunity to introduce structured postgraduate training in diabetes.

## Abbreviations

CR: Confidence Rating; UK: United Kingdom of Great Britain and Northern Ireland.

## Competing interests

The author(s) declare that they have no competing interests.

## Authors' contributions

JTG came up with the concept for this research projects. JTG, GAM and EBJ designed the questionnaire with input from SX and JA. JTG, KSR and DW undertook the literature review; SX, JTG and EBJ administered the questionnaire. DW, JTG and GM. DW, KSR, JTG carried out data analysis. JTG and GAM drafted this manuscript, which was revised by EBJ. All authors have read and approved the manuscript.

## Pre-publication history

The pre-publication history for this paper can be accessed here:



## References

[B1] King H, Aubert R, Herman W (1998). Global burden of diabetes, 1995–2025: Prevalence, numerical estimates, and projections. Diabetes Care.

[B2] Diabetes UK State of the nations 2006. http://www.diabetes.org.uk.

[B3] York & Humber Public Health Observatory (2006). Diabetes KeyFacts National Diabetes Support Team.

[B4] (1990). UK prospective diabetes study 6. complications in newly diagnosed type 2 diabetic patients and their association with different clinical and biochemical risk factors. Diabetes Res.

[B5] Lasker RD (1993). The diabetes control and complications trial – implications for policy and practice. N Engl J Med.

[B6] Donnan PT, MacDonald TM, A. D. Morris for the DARTS/MEMO Collaboration (2002). Adherence to prescribed oral hypoglycaemic medication in a population of patients with type 2 diabetes: A retrospective cohort study.[article]. Diabetic Med.

[B7] Garber AJ (2003). Benefits of combination therapy of insulin and oral hypoglycemic agents.[miscellaneous]. Arch Intern Med.

[B8] Stratton IM, Adler AI, Neil HAW, Matthews DR, Manley SE, Cull CA, Hadden D, Turner RC, Holman RR (2000). Association of glycaemia with macrovascular and microvascular complications of type 2 diabetes (UKPDS 35): Prospective observational study. BMJ.

[B9] Colhoun HM, Betteridge DJ, Durrington PN, Hitman GA, Neil HA, Livingstone SJ, Thomason MJ, Mackness MI, Charlton-Menys V, Fuller JH, CARDS investigators (2004). Primary prevention of cardiovascular disease with atorvastatin in type 2 diabetes in the collaborative atorvastatin diabetes study (CARDS): Multicentre randomised placebo-controlled trial. Lancet.

[B10] American Diabetes Association (2007). Standards of medical care in diabetes – 2007. Diabetes Care.

[B11] IDF Task Force on Clinical Practice Guidelines (2005). Global Guideline for Type 2 Diabetes.

[B12] Drazen JM, Morrissey S, Curfman GD (2007). Rosiglitazone – continued uncertainty about safety. N Engl J Med.

[B13] Baldwin D, Villanueva G, McNutt R, Bhatnagar S (2005). Eliminating inpatient sliding-scale insulin: A reeducation project with medical house staff. Diabetes Care.

[B14] Modernising medical careers. http://www.mmc.nhs.uk.

[B15] Appraisal record for senior house officers. http://www.rcplondon.ac.uk/pubs/contents/304a5fdc-cc4e-4d3e-8270-49403e35fe8e.pdf.

[B16] The ACE/ADA Task Force on Inpatient Diabetes (2006). American college of endocrinology and american diabetes association consensus statement on inpatient diabetes and glycemic control: A call to action. Diabetes Care.

[B17] Van den Berghe G, Wouters P, Weekers F, Verwaest C, Bruyninckx F, Schetz M, Vlasselaers D, Ferdinande P, Lauwers P, Bouillon R (2001). Intensive insulin therapy in critically ill patients. N Engl J Med.

[B18] Beckley ET (2005). IDF issues global type 2 diabetes guidelines. DOC News.

[B19] Wallymahmed ME, Dawes S, Clarke G, Saunders S, Younis N, MacFarlane IA (2005). Hospital in-patients with diabetes: Increasing prevalence and management problems. Diabetic Med.

[B20] Sampson MJ, Brennan C, Dhatariya K, Jones C, Walden E (2007). A national survey of in-patient diabetes services in the united kingdom. Diabet Med.

[B21] Jefferson IG, Swift PG, Skinner TC, Hood GK (2003). Diabetes services in the UK: Third national survey confirms continuing deficiencies. Arch Dis Child.

[B22] Department of Health (2003). National service framework for diabetes: Standards. London.

[B23] Hessett C, Moran A, Boulton AJ (1989). An evaluation of diabetes knowledge amongst general practitioners and senior medical students. central manchester health authority working party on diabetes care. Diabet Med.

[B24] Goldacre MJ, Lambert T, Evans J, Turner G (2003). Preregistration house officers' views on whether their experience at medical school prepared them well for their jobs: National questionnaire survey. BMJ.

[B25] MMC Inquiry Panel Aspiring for Excellence -Final Report of the independent inquiry into Modernising Medical Careers. http://www.mmcinquiry.org.uk/MMC_FINAL_REPORT_REVD_4jan.pdf.

